# The Effect of the Pre-Strain Process on the Strain Engineering of Two-Dimensional Materials and Their van der Waals Heterostructures

**DOI:** 10.3390/nano13050833

**Published:** 2023-02-23

**Authors:** Jinkun Han, Xiaofei Yue, Yabing Shan, Jiajun Chen, Borgea G. M. Ekoya, Laigui Hu, Ran Liu, Zhijun Qiu, Chunxiao Cong

**Affiliations:** 1 State Key Laboratory of ASIC and System, School of Information Science and Technology, Fudan University, Shanghai 200433, China; 2 Yiwu Research Institute of Fudan University, Chengbei Road, Yiwu City 322000, China

**Keywords:** van der Waals heterostructures, strain, transition metal dichalcogenides, photoluminescence

## Abstract

Two-dimensional (2D) materials and their van der Waals stacked heterostructures (vdWH) are becoming the rising and glowing candidates in the emerging flexible nanoelectronics and optoelectronic industry. Strain engineering proves to be an efficient way to modulate the band structure of 2D materials and their vdWH, which will broaden understanding and practical applications of the material. Therefore, how to apply desired strain to 2D materials and their vdWH is of great importance to get the intrinsic understanding of 2D materials and their vdWH with strain modulation. Here, systematic and comparative studies of strain engineering on monolayer WSe_2_ and graphene/WSe_2_ heterostructure are studied by photoluminescence (PL) measurements under uniaxial tensile strain. It is found that contacts between graphene and WSe_2_ interface are improved, and the residual strain is relieved through the pre-strain process, which thus results in the comparable shift rate of the neutral exciton (A) and trion (A^T^) of monolayer WSe_2_ and graphene/WSe_2_ heterostructure under the subsequent strain release process. Furthermore, the PL quenching occurred when the strain is restored to the original position also indicates the pre-strain process to 2D materials, and their vdWH is important and necessary for improving the interface contacts and reducing the residual strain. Thus, the intrinsic response of the 2D material and their vdWH under strain can be obtained after the pre-strain treatment. These findings provide a quick, fast and efficient way to apply desired strain and also have important significance in guiding the use of 2D materials and their vdWH in the field of flexible and wearable devices.

## 1. Introduction

Due to the unique optical, electrical and mechanical properties, two-dimensional (2D) materials and their heterostructures have drawn great attention in the field of semiconductor technology along with the emerging flexible nanoelectronics and optoelectronics. Thickness-dependent properties of 2D materials also open possibilities in the fabrication of devices in the fields of semiconductors, insulators, transparent conductors and sensors [1,2,3,4,5]. Combined with polymer or plastic substrates [6,7,8], flexible nanodevices based on 2D materials exhibit good device performance together with excellent mechanical properties, which makes the long-term pursuit of large-scale manufacturing of high-performance flexible devices become a realistic possibility [9,10,11].

Among the 2D materials, TMDs (transition metal dichalcogenide) in two-dimension with direct bandgap are good candidates for flexible nanoelectronics and optoelectronic devices [12,13,14,15,16]. Besides, heterostructures formed through the inter-layer vdWH force between 2D materials exhibit superior photoelectric characteristics because of the internal charge-limited movement due to interlayer charge transfer [17,18,19,20,21], leading to promising applications in optoelectronics.

Previous studies have shown that strain engineering is an effective way to tune the properties of 2D materials such as electronic band structures, sub-effective mass, conductivity, exciton-phonon coupling, and spin-orbit coupling by changing the lattice structure of 2D materials (such as bond length, bond angle, the relative position of atoms and lattice symmetry) [22,23,24,25,26,27]. However, efficiently applying desired strain to the sample is challenging due to non-uniform interfaces between a sample and the flexible substrate, the weakly bonded nature of the van der Waals stacked heterostructures (vdWH) and residual strain induced during the sample fabrication process. It has been demonstrated that the annealing process during the sample and device fabrication procedure plays an important role in obtaining good interfaces within the vdWH and the interface between the sample and substrate [28,29,30]. However, the organic nature of flexible substrates makes them unsuitable for the annealing process. It is important and necessary to find a fast, non-destructive and efficient way to exert desired strain on 2D materials and their vdWH efficiently.

Here, we provide a pre-strain method to efficiently exert desired strain on the 2D materials and their vdWH on the flexible substrates. We first performed a systematic and comparative study about strain modulation on a monolayer (1L) WSe_2_ and graphene/WSe_2_ heterostructure by applying reversible uniaxial strain. The larger shift rate difference of A and A^T^ between monolayer WSe_2_ and graphene/WSe_2_ heterostructure during the strain on process may be due to the incomplete contact between graphene and WSe_2_ interface and the residual strain introduced during the heterostructure fabrication process. After the strain on process (pre-strain), contacts between the graphene and WSe_2_ interface were greatly improved, and the residual strain was released, which thus resulted in the comparable shift rate of monolayer WSe_2_ and graphene/WSe_2_ heterostructure under the subsequent strain release process. The almost same A and A^T^ changing rate of monolayer WSe_2_ and graphene/WSe_2_ heterostructure during the strain release process clearly shows that pre-strain treatment is an easy and fast way to exert desired strain on the as-prepared sample. Furthermore, the improvement of contacts between graphene and WSe_2_ interface and the release of residual strain can also be manifested by the quenching phenomenon after the strain is released to the original position and more uniformity of the A and A^T^ emission energies showing by photoluminescence (PL) mapping after strain on and release process. To further confirm the pre-strain effect, we also studied the pre-strain effect on the strain modulation for monolayer WS_2_, WSe_2_/WS_2_ heterostructure and MoSe_2_/WS_2_ heterostructure. It is found that pre-strain can well improve the interface and the contacts as well as release the residual strain between 2D materials and/or 2D materials and substrates. Therefore, the pre-applied strain process is important and necessary in strain engineering of 2D materials and their vdWH, which also gives important guides to the practical applications of 2D materials and their vdWH in flexible devices.

## 2. Results and Discussion

**Figure 1:**
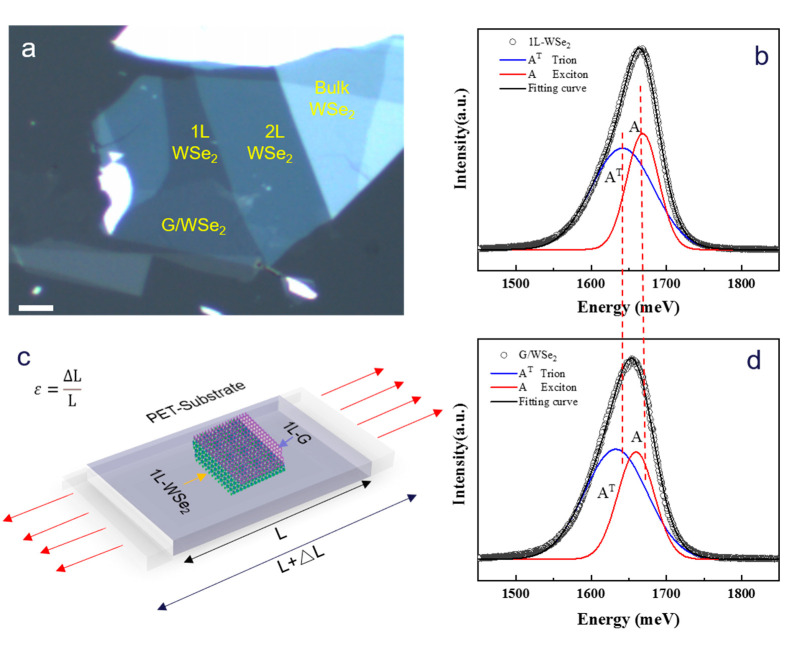
(**a**) Optical image of fabricated monolayer WSe_2_ and graphene/WSe_2_ heterostructure on PET substrate. The scale bar is 4μm. (**b**) PL spectrum with Gauss fitting for 1L WSe_2_. (**c**) Schematic illustration of the uniaxial tensile strain we applied in this work. (**d**) PL spectrum with Gauss fitting for 1L graphene/WSe_2_ heterostructure. The red dot line is the eye-guide of clearly read shift for both Exciton A and Trion A^T^ peak in graphene/WSe_2_ heterostructure compared to 1L WSe_2_.

We performed systemic strain modulation (refer to Strain Modulation Section for detail) on monolayer WSe_2_, and monolayer graphene/Wse_2_ heterostructure on polyethene terephthalate (PET) (refer to Material Fabrication Section for detail) substrate with revisable uniaxial tensile strain ranged from 0 to 2.4%, Figure 1a is the optical image of fabricated monolayer WSe_2_ and graphene/WSe_2_ heterostructure on PET substrate, in which different areas are labelled on the sample and can be clearly distinguished from each other. Figure 1c shows the schematic illustration of the uniaxial tensile strain we applied in this work. The in-situ strain-dependent PL measurements with revisable uniaxial tensile strain ranging from 0 to 2.4% are carried out to probe the strain modulation on the properties of 1L WSe_2_ and 1L graphene/WSe_2_ heterostructure (refer to Measurement section for detail). Both the PLspectrum of monolayer WSe_2_ and graphene/WSe_2_ heterostructure consists of two components, corresponding to the neutral exciton (A) and trion (A^T^), respectively. The energy difference between A and A^T^ in 1L-WSe_2_ and graphene/WSe_2_ heterostructure are both around ~31 meV (shown in Figure 1a,b), which matches well with previous studies [31]. Compared to monolayer WSe_2_, the PL emission of graphene/WSe_2_ heterostructure shows a red shift (Figure 1b,d). This may be attributed to the charge transfer between monolayer graphene and WSe_2_ and/or the residual strain and impurities introduced during the heterostructure fabrication process.

**Figure 2:**
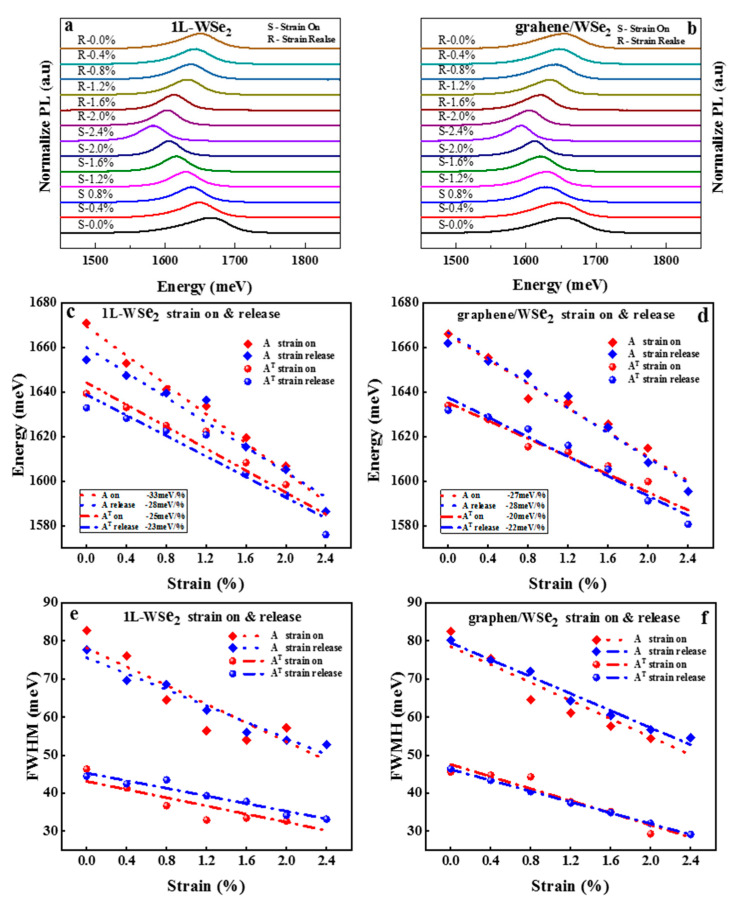
(**a**) PL spectra with strain on and release for 1L WSe_2_. (**b**) PL spectra with strain on and re-lease for graphene/WSe_2_ heterostructure. (**c**) The fitted PL emission energies of A and A^T^ peaks in 1L WSe_2_ with the strain on and release process from 0 to 2.4%. (**d**) The fitted PL emission energies of A and A^T^ peaks in graphene/WSe_2_ heterostructure with the strain on and release process from 0 to 2.4%. (**e**) The fitted FWMH of A and A^T^ peaks in 1L WSe_2_ with the strain on and release process from 0 to 2.4%. (**f**) The fitted FWMH of A and A^T^ peaks in graphene/WSe_2_ with the strain on and release process from 0 to 2.4%.

Figure 2a,b shows the PL spectra of monolayer WSe_2_ and graphene/WSe_2_ heterostructure with strain on (0.0–2.4%) and strain release (2.4–0.0%) process, respectively. It can be seen that an obvious red shift of PL emission energies for both monolayer WSe_2_ and graphene/WSe_2_ heterostructure is observed during strain on process versus blue shift during the strain release process, which indicates the uniaxial tensile strain can well-modulated the band structure of monolayer WSe_2_ and graphene/WSe_2_ heterostructure. To further prove the effect of pre-strain, the PL spectra of monolayer WSe_2_ and graphene/WSe_2_ heterostructure are fitted as two Gaussian peaks (A (exciton) and A^T^ (trion)). The fitted light emission energies as a function of strain during the strain on and release process are shown in Figure 2c,d. Figure 2e,f shows the fitted full-width-at-half maximum (FWHM) as a function of strain during strain on and release process accordingly. Figure 2c,d show that during strain on the process, red shift gauge factor values of A peak for monolayer WSe_2_ and graphene/WSe_2_ heterostructure are 33 meV per 1% strain (33 meV/%) and 27 meV/%, while the values of A^T^ peaks are 25 meV/% and 20 meV/%. When the strain is released, the A and A^+^ peak of the monolayer WSe_2_ and the graphene/WSe_2_ heterostructure is blue shifted with 28 meV/% (A of WSe_2_), 28 meV/% (A of graphene/WSe_2_), 23 meV/% (A^T^ of WSe_2_), and 22 meV/% (A^T^ of graphene/WSe_2_). The changing rate of light emission energies for A and A^T^ during the release process of the heterostructure is closer to that of 1L WSe_2_, indicating contacts between graphene/WSe_2_ and WSe_2_/substrate are well improved and the possibility that the residual strain during the sample fabrication process is well released after the strain on process (namely pre-strain). Besides, the fitting linearity of A and A^T^ during the strain release process is much smoother than that during the strain on (pre-strain) process, which also can be drawn from Figure 2c,d. Moreover, the changing fluctuations of FWHM of PL peak for monolayer WSe_2_ and graphene/WSe_2_ heterostructure during strain on and release process is shown in Figure 2e,f also highlights the above conclusion. Therefore, compared with strain on the process, changing fluctuations of PL peak energies and FWHM for both monolayer WSe_2_ and graphene/WSe_2_ heterostructure is relatively small during the strain release process, thus resulting in a much smoother overall changing trend after the pre-stain process.

**Figure 3:**
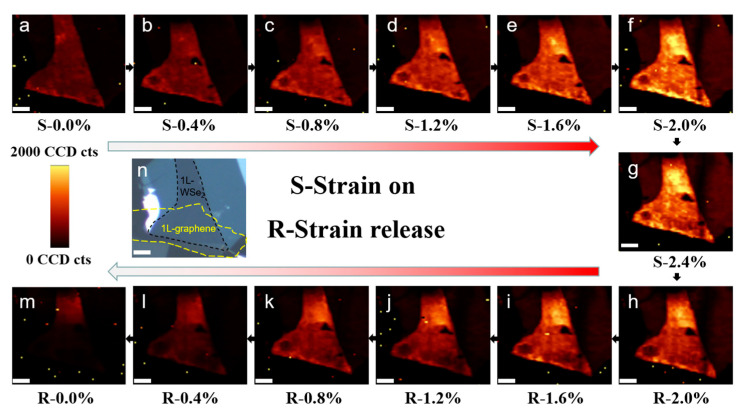
(**a**–**g**) corresponds to PL emission intensity of monolayer WSe_2_ and graphene/WSe_2_ heterostructure for the strain on the process from 0% to 2.4%, (**g**–**m**) corresponds to PL emission intensity of monolayer WSe_2_ and graphene/WSe_2_ heterostructure for strain release process from 2.4% to 0%. (**n**) is the optical view showing the boundary of the sample; the black area is the bottom layer 1L-WSe_2_, and the yellow area is the top layer 1L-graphene. The scale bar is 4 μm in all figures.

To further reveal the effect of pre-strain, the PL mapping measurements under strain modulation are performed for the monolayer WSe_2_ and graphene/WSe_2_ heterostructure since PL mapping can directly embody the overall change of the sample (refer to Figure S3 for an optical view of the sample under each strain value). Figure 3a–g shows the PL emission intensity of monolayer WSe_2_ and graphene/WSe_2_ heterostructure for the strain on the process from 0% to 2.4%, while the PL emission intensity of monolayer WSe_2_ and graphene/WSe_2_ heterostructure for strain release process from 2.4% to 0% is shown in Figure 3g–m. All the mapping images of PL emission intensity in Figure 3 keep the same intensity scale. Obviously, after pre-strain, the PL emission intensity of graphene/WSe_2_ heterostructure is decreased gradually during the strain release process and quenched when strain release to 0.0% (Figure 3g–m). Whereas the PL emission intensity of monolayer WSe_2_ does not change obviously during the strain release process. Thus, compared to the counterpart strain on the process (Figure 3a–f), the contrast of the PL emission intensity between the monolayer WSe_2_ part and graphene/WSe_2_ heterostructure part is more and more obvious (Figure 3h–m), which prove interlayer contacts are well improved after straining on the process. It is known that PL quenching can be used as an indicator of fine contact between WSe_2_ and graphene [32]. The PL quenching (Figure 3m) happened when strain release to 0.0% further proved the improvement of contact between graphene and WSe_2_ interface and the release of residual strain after pre-strain treatment.

**Figure 4:**
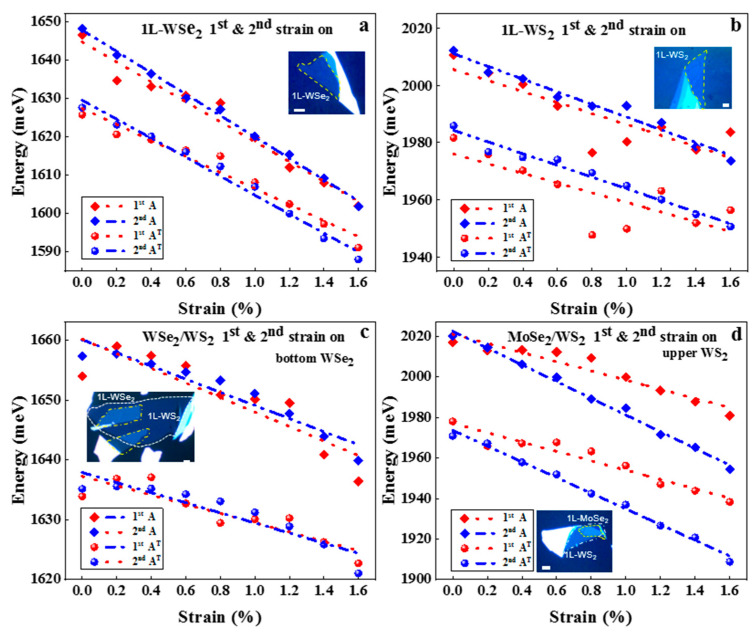
The fitted PL emission energies of A and A^T^ peaks in 1L-WSe_2_ (**a**) and A and A^T^ peaks in 1L-WS_2_ (**b**) with the increasing strain from 0 to 1.6% for the first strain on (pre-strain) and the second strain on process. Insert is the optical image of the 1L WSe_2_ (**a**) and 1L-WS_2_ (**b**) (yellow marked area). The fitted PL emission energies of A and A^T^ peaks of bottom 1L-WSe_2_ in WSe_2_/WS_2_ heterostructure (**c**) and A and A^T^ peaks of upper 1L-WS_2_ in MoSe_2_/WS_2_ heterostructure (**d**) with the increasing strain from 0 to 1.6% for the first strain on (pre-strain) and the second strain on process. Insert is the optical image of the WSe_2_/WS_2_ (**c**) (yellow frame 1l-WSe_2_ and white frame 1L-WS_2_) and MoSe_2_/WS_2_ (yellow frame 1l-MoSe_2_ and white frame 1L-WS_2_) (**d**) heterostructure. The scale bar is 4 μm in all figures.

To further confirm our conclusion, we applied pre-strain treatment to all the samples shown in Figure 4. For each sample in Figure 4, we first applied strain up to 1.6%, then released it to 0% and re-do the strain modulation again. Figure 4a shows the change of fitted PL emission energies (A and A^T^) of 1L-WSe_2_ during the first (pre-strain) and second strain on process. It shows that the A and at the peak of 1L-WSe_2_ during the second strain on the process are much smoother, and the fitting curves are more linear, which can be attributed to the same reasons as monolayer WSe_2_ in Figure 1a. Figure 4b is the change of fitted PL emission energies A and A^T^) for 1L-WS_2_ during the first (pre-strain) and second strain modulation process, in which the fluctuations during the pre-strain process show poor interface contacts and/or large residue strain between WS_2_ and PET substrate and thus improved during the second strain on process. Figure 4c,d shows the strain modulation for the heterostructures of WSe_2_/WS_2_ and MoSe_2_/WS_2_ with pre-strain treatment. Figure 4c shows the fitted PL emission energies of A and A^T^ peaks of bottom monolayer WSe_2_ in WSe_2_/WS_2_ heterostructure with the strain from 0 to 1.6% during the first (pre-strain) and second strain on process. In this structure, WSe_2_ is sandwiched between PET and WS_2,_ thus resulting in the related small change of the fitted PL emission energies fitting curve, but interface contacts improved after the pre-strain process, which makes fluctuations smoother compared to the pre-strain process. Figure 4d shows the fitted PL emission energies of A and A^−^ peaks of upper monolayer WS_2_ in MoSe_2_/WS_2_ heterostructure with the strain from 0 to 1.6% for the first (pre-strain) and second strain on process. Generally, strain needs to be transferred from the lower layer to the upper layer, indicating that strain can be effectively transferred between layers. Meanwhile, the pre-strain process improves contacts between the substrate and different layers, thus making strain transfer to the upper layer more efficient. The slope of the fitting curve for the second strain on process is higher than that for the first strain on process, as shown in Figure 4d. All the samples above show the importance of the pre-strain and give a clear illustration of the importance and necessity of the pre-strain process in strain modulation of 2D materials and give guidance to the real strain-related applications of 2D materials.

## 3. Conclusions

In summary, we have systematically investigated the effect of pre-strain modulation on the strain engineering of monolayer WSe_2_ and graphene-WSe_2_ heterostructures. The results show that pre-strain can well improve the interface contacts and release residual strain between materials and/or materials and substrates, reducing impurity doping and defects introduced during the sample fabrication process. Considering the temperature condition required in annealing is hard to achieve for PET and another flexible substrate, which is usually organic. The pre-strain process improves contacts and reduces doping and impurity during the sample fabrication process easily, effectively and non-destructively. Therefore, these findings point out a simple, fast and effective way to obtain intrinsic properties of 2D materials and their vdWH under strain engineering, which gives a basic rule and requirement for the application of strain engineering on two-dimensional materials and their heterostructures in both lab experiments and real nano-flexible industry.

## 4. Experimental

### 4.1. Material Fabrication

Monolayer WSe_2_ and monolayer graphene are exfoliated onto the PET and PDMS (refer to Figure S1 for Raman spectrum) substrate by micro-mechanical exfoliation method, respectively. Then by using a deterministic transfer stage with translation stage of X, Y, and Z axis equipped under an optical microscope, the monolayer graphene on PDMS was transferred to the top of the monolayer WSe_2_ on PET to form graphene/WSe_2_ heterostructure (dry transfer method). Other samples shown in Figure 4 are all fabricated by the above method.

### 4.2. Strain Modulation

The samples supported on PET substrate are fixed onto the strain stage by glue; thus, the in-situ Raman and PL measurements can be conducted under controllable strain. Uniaxial tensile strain is applied with a translation stage by rotating the micrometre of the stage to elongate the PET substrate in the horizontal direction, as shown in Figure 1c. The applied strain is calculated through the ratio between the elongated length (ΔL) and the original length (L = 5 mm in this experiment). The micrometre of the translation stage moved with the step of 0.02 mm to achieve the desired strain values with the step of 0.4% (refer to Figure S2 for schematic illustration).

### 4.3. Measurement

The Raman and PL spectra in the experiments are collected by the confocal Raman system (WITec Alpha300R) with 1800 lines/mm grating and 600 lines/mm grating, respectively. A 100× objective lens with a numerical aperture (N.A.) of 0.95 was used for both PL and Raman measurements. The laser spot size is about 500 nm in diameter. The excitation laser wavelength for both Raman and PL is 532 nm. The laser power was set to 0.5 mw for Raman measurements and 0.01 mW for PL measurements to avoid damage and potential heating effect on the sample.

## Data Availability

The authors confirm that the data supporting the findings of this study are available within the article and its supplementary materials.

## Supplementary Materials

The following supporting information can be downloaded at: https://www.mdpi.com/article/10.3390/nano13050833/s1, Figure S1: Raman spectrum; Figure S2: Strain modulation schematic; Figure S3: Optical view of 1L-WSe_2_ and graphene/WSe_2_ heterostructure.
