# Supplementary material for: The Effect of the Pre-Strain Process on the Strain Engineering of Two-Dimensional Materials and Their van der Waals Heterostructures
Source: Nanomaterials (Basel). 2023 Feb 23;13(5):833. doi: 10.3390/nano13050833 (PMC10005575; doi:10.3390/nano13050833)
Supplement: Supplementary file 1 [file nanomaterials-13-00833-s001.zip › nanomaterials-2171902-supplementary.pdf]

The Effect of the Pre-Strain Process on the Strain Engineering of Two-Dimensional Materials and Their van der Waals Heterostructures

Jinkun Han [1], Xiaofei Yue [1], Yabing Shan [1], Jiajun Chen [1], Borgea G. M. Ekoya [1], Laigui Hu [1], Ran Liu [1], Zhijun Qiu [1,*] and Chunxiao Cong [1,2,*]

[1] State Key Laboratory of ASIC and System, School of Information Science and Technology, Fudan University, Shanghai 200433, China

[2] Yiwu Research Institute of Fudan University, Chengbei Road, Yiwu City 322000, China

* Correspondence: zjqiu@fudan.edu.cn (Z.Q.); cxcong@fudan.edu.cn (C.C.)

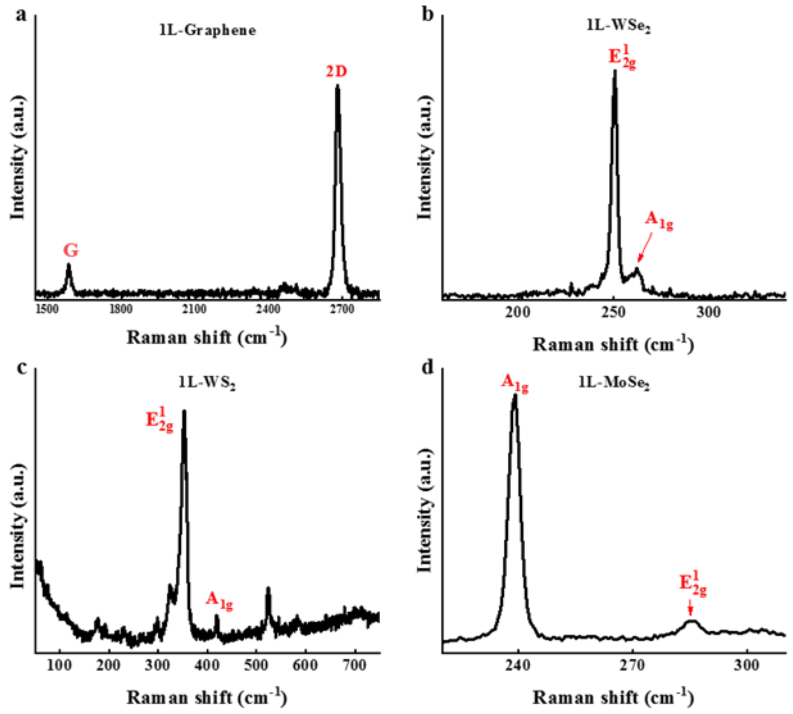

Figure S1. (a–d) Raman spectrum for 1L-graphene, 1L-WSe_2,1L-WS_2,1L-$MoSe_2$.

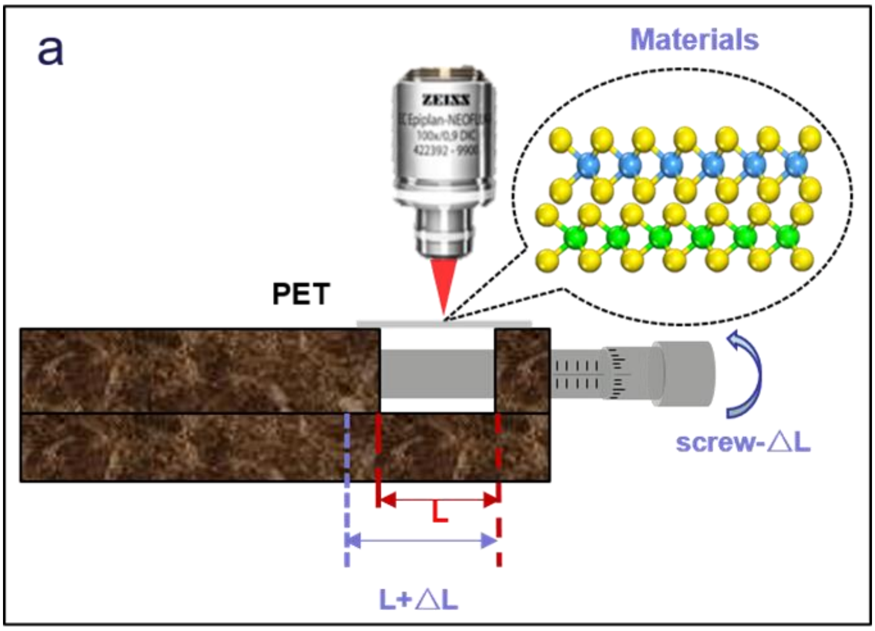

Figure S2. (a) Strain modulation schematic for WS_2/WSe_2 and $WS_2/MoSe_2$ heterostructure.

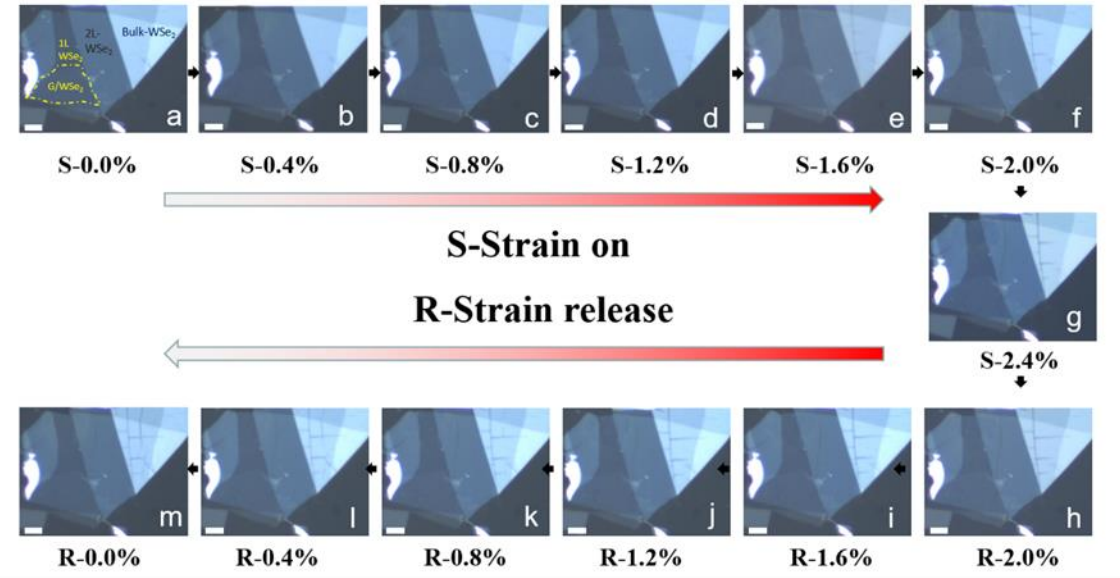

Figure S3. (a–m) Optical view of 1L-WSe_2 and graphene/WSe_2 heterostructure during strain on and release process from 0–2.4%.
